# Automated assessment of functional lung imaging with ^68^Ga-ventilation/perfusion PET/CT using iterative histogram analysis

**DOI:** 10.1186/s40658-021-00375-6

**Published:** 2021-03-07

**Authors:** Lachlan McIntosh, Price Jackson, Nicholas Hardcastle, Mathias Bressel, Tomas Kron, Jason W. Callahan, Daniel Steinfort, Nicholas Bucknell, Michael S. Hofman, Shankar Siva

**Affiliations:** 1 Department of Physical Sciences, Peter MacCallum Cancer Centre, Melbourne, 3000 Australia; 2 Department of Cancer Imaging, Peter MacCallum Cancer Centre, Melbourne, 3000 Australia; 3 Sir Peter MacCallum Department of Oncology, University of Melbourne, Melbourne, 3010 Australia; 4 Centre for Medical Radiation Physics, University of Wollongong, Wollongong, New South Wales 2522 Australia; 5 Centre for Biostatistics and Clinical Trials, Peter MacCallum Cancer Centre, Melbourne, Victoria Australia; 6 Respiratory Medicine, Peter MacCallum Cancer Centre and Royal Melbourne Hospital, Melbourne, Australia; 7 Department of Radiation Oncology, Peter MacCallum Cancer Centre, Melbourne, 3000 Australia

**Keywords:** V/Q PET/CT, Gallium 68, Regional lung function, Delineation

## Abstract

**Purpose:**

Functional lung mapping from Ga^68^-ventilation/perfusion (V/Q) PET/CT, which has been shown to correlate with pulmonary function tests (PFTs), may be beneficial in a number of clinical applications where sparing regions of high lung function is of interest. Regions of clumping in the proximal airways in patients with airways disease can result in areas of focal intense activity and artefact in ventilation imaging. These artefacts may even shine through to subsequent perfusion images and create a challenge for quantitative analysis of PET imaging. We aimed to develop an automated algorithm that interprets the uptake histogram of PET images to calculate a peak uptake value more representative of the global lung volume.

**Methods:**

Sixty-six patients recruited from a prospective clinical trial underwent both V/Q PET/CT imaging and PFT analysis before treatment. PET images were normalised using an iterative histogram analysis technique to account for tracer hotspots prior to the threshold-based delineation of varying values. Pearson’s correlation between fractional lung function and PFT score was calculated for ventilation, perfusion, and matched imaging volumes at varying threshold values.

**Results:**

For all functional imaging thresholds, only FEV1/FVC PFT yielded reasonable correlations to image-based functional volume. For ventilation, a range of 10–30% of adapted peak uptake value provided a reasonable threshold to define a volume that correlated with FEV1/FVC (*r* = 0.54–0.61). For perfusion imaging, a similar correlation was observed (*r* = 0.51–0.56) in the range of 20–60% adapted peak threshold. Matched volumes were closely linked to ventilation with a threshold range of 15–35% yielding a similar correlation (*r* = 0.55–0.58).

**Conclusions:**

Histogram normalisation may be implemented to determine the presence of tracer clumping hotspots in Ga-68 V/Q PET imaging allowing for automated delineation of functional lung and standardisation of functional volume reporting.

## Introduction

Functional lung volume obtained from Ga^68^-ventilation/perfusion (V/Q) PET/CT imaging, a fractional measure of the total lung that exhibits appreciable uptake of inhaled or perfused tracer, has been shown to correlate with pulmonary function tests (PFTs) [1, 2]. This shows promise in utilising volumetric imaging to determine areas which may warrant avoidance in external beam radiotherapy [3, 4], assessment of radiation injury to the lung [5], and estimating the loss of function after surgery in the context of resection of lung cancers [6–8]. In previous work, functional volumes were assessed visually to determine appropriate uptake thresholds and manually corrected where necessary. This process is laborious, requires clinician input, and is subject to interobserver variability. Previously, an appropriate threshold to define a functional volume was adapted to each case, and in some instances, manual removal of non-physiological uptake was required [2].

As with Technegas, clumping of radiotracer is sometimes observed in the proximal airways with Galligas. More artefact is typical in patients with severe airway disease, and areas of uptake have focal intense uptake which interferes with quantitative analysis. As the perfusion acquisition is obtained shortly after the ventilation acquisition, “shine through” of these high count areas may occur, also resulting in artefact on the perfusion images. The aim of this work was to identify an automated, objective means to improve on the previous process by implementing an iterative normalisation of the maximum uptake voxel intensity prior to thresholding—an initialisation step to remove the requirement for manual pre-processing by the reviewer.

## Methods

### Patients

Sixty-six patients (43 males, 23 females; mean age 66 years, range 34–89) with stage III non-small cell lung cancer planned to receive radiation therapy with curative intent were analysed. Patients had ventilation/perfusion PET/CT and pulmonary function tests, as part of a study assessing lung function from radiotherapy (Australian New Zealand Clinical Trial Registry Trial ID 12613000061730). The study was approved by the Peter MacCallum Clinical Governance and Ethics Committee. The study design and patient cohort have been comprehensively described in a previous paper [9].

### Pulmonary function tests

All patients underwent PFT assessment prior to radiation therapy according to forced vital capacity (FVC), forced expiratory volume (FEV), FEV/FVC ratio, and carbon monoxide diffusing capacity (DLCO). Mean baseline PFT assessments (actual/predicted) FVC, FEV, FEV/FVC ratio, and DLCO were 91%, 76%, 65%, and 64%, respectively. PFTs ranged from 47 to 134%, 31 to 121%, 30 to 90%, and 23 to 102% for FVC, FEV, FEV/FVC, and DLCO, respectively.

### ^68^Ga-V/Q PET/CT protocol

Ventilation PET imaging was acquired via inhalation of ^68^Ga carbon nanoparticles (Galligas) [10] followed by an injection of approximately 50 MBq of ^68^Ga-labelled macroaggregated albumin (MAA) for perfusion imaging. In 14 of 66 cases, only a perfusion study was performed due to tracer availability. PET studies were performed over two bed positions where each bed position was acquired for 5 min. The protocol for ^68^Ga-V/Q imaging has previously been described previously [11, 12].

### Functional volume delineation

Functional volume delineation can be summarised in three steps: whole lung uptake characterisation, iterative reduction in outlier uptake values via histogram normalisation, and subsequent thresholding based on the converged peak intensity uptake value. To characterise whole lung uptake, PET voxels were limited to a lung contour that was defined based on CT margins. The converged peak intensity value is an iteratively reduced uptake value based on a histogram analysis of uptake inside the lung contour. The purpose of this step is to anchor threshold values to a more accurate value compared to an overall maximum and addresses non-physiological artefacts in both ventilation and perfusion imaging such as Galligas clumping in the airways.

### Histogram normalisation using iterative convergence

**Fig. 1:**
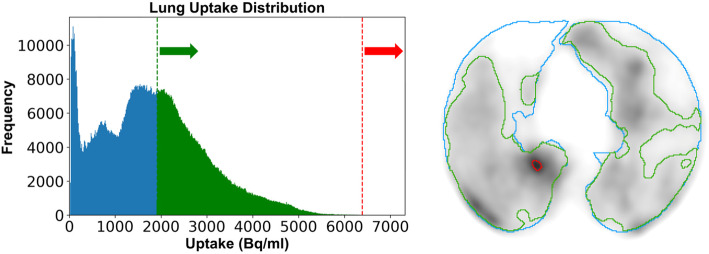
Example of functional volume delineation in the presence of an airway artifact. Whole lung volume is shown in blue. The aggregated, non-physiological uptake (red) is detected by histogram analysis, and a more representative peak uptake value is designated. The resultant functional volume from 30% threshold is shown in green

In order to remove the requirement for manual corrections of artefacts in ventilation imaging via airway clumping and perfusion imaging via MAA clumping, a reduction in the maximum uptake voxel was computationally implemented. The maximum intensity measured in the whole lung uptake was iteratively reduced to a new value (mean + 4 standard deviations) until a difference between the calculated value and previous maximum was less than 1%. In situations where the initial iteration calculates a value within 1% of the original maximum or greater than the original maximum, no reduction in maximum intensity was applied. An example of the reduced maximum intensity anchoring threshold is given in Fig. 1. This accounts for very high outlier values which otherwise skew the uptake distribution histogram of intensities within the lung and yields a peak intensity value which is in closer accordance with the uptake distribution within high functioning lung tissue. This iterative technique is similar to that used by Kipritidis et al. for clumping hotspot removal [13] and was implemented using Python 3.7 with libraries SimpleITK and NumPy.

Iterative maximum intensity reduction was considered to be converged when the convergence parameter (*z*) was greater than 99% (i.e. the step-wise value less than 1% of the previous). The first iteration step (*i* = 1) determined if the maximum intensity reduction was required at all. For example, if the initial calculated mean + 4 standard deviations inside the lung were within 1% of the original maximum, no reduction was applied and the original maximum was considered to be a representative maximum. The resolved peak intensity is a function of the mean (*x̄*) and standard deviation (*σ*) uptake inside the lung. Volumes above the peak at each step are ignored in successive iterations but are reintroduced into the final volume, reduced to the new peak intensity.

$$ {z}_{\left[i>1\right]}=\frac{{\left(x\bar +4\sigma \right)}_i}{{\left(x\bar +4\sigma \right)}_{i-1}} $$

$$ {\mathrm{peak}\ \mathrm{intensity}}_{\left[z\to 0.99\right]}={\left(x\bar +4\sigma \right)}_i $$

### Thresholding

A range of threshold values (5–70% of the peak intensity, in 5% increments) were applied to each ventilation and perfusion image after the normalisation process to investigate an optimal functional correlation across the cohort. A fractional value was given to each image at each threshold, defining the fraction of functional lung volume delineated relative to total lung volume. Where both ventilation and perfusion imaging data was available (*n* = 52/66), a matched functional volume was generated using the intersection of functional volumes in both images at the same threshold. Functional volumes from all of the thresholds were then correlated against pulmonary function tests: FVC (%Predicted), FEV (%Predicted), FEV/FVC, and DLCO (%Predicted)—using Pearson correlation.

## Results

Peak uptake normalisation was more frequently required in ventilation imaging (48/52) than perfusion imaging (21/66). Relative volumes normalised for ventilation and perfusion imaging were 0.23% and 0.06%, respectively. For all studies, across both imaging techniques, the maximum volume normalised was 4.88% in a perfusion image—as shown in Table 1.

**Table 1:** Median, mean, and max volumes relative to the total lung that were normalised

Imaging technique	Volume normalised (% relative to total lung)		
	Median	Mean	Max
Ventilation (*n* = 48/52)	0.23%	0.53%	3.34%
Perfusion (*n* = 21/66)	0.06%	0.51%	4.88%

**Fig. 2:**
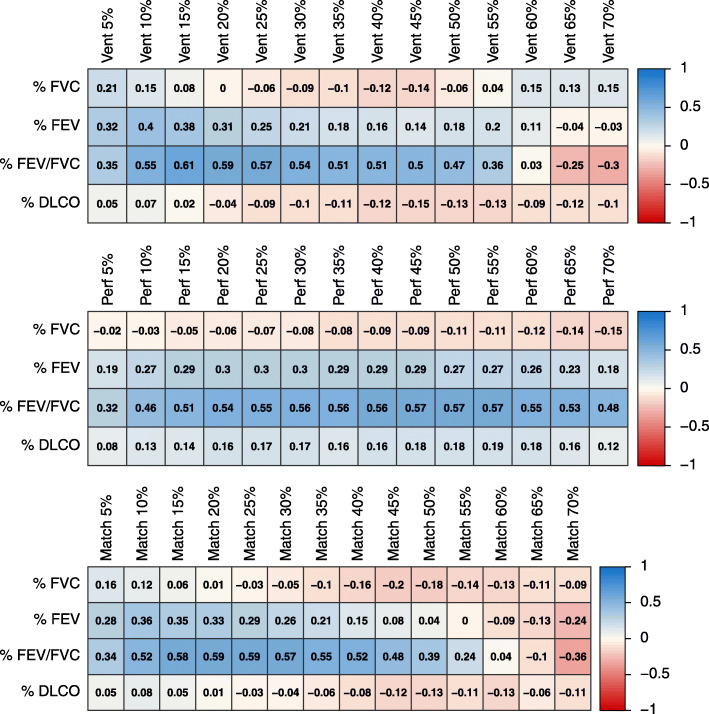
Correlogram of baseline volumes vs. baseline PFT

Correlograms illustrating the correlation of lung function measures and resolved peak intensity threshold for each imaging technique are given in Fig. 2. The range of correlations plateaus between threshold minimum and maximum boundaries for each volume. For all PFTs correlated against functional volumes, FEV/FVC consistently yielded the greatest correlation to PET-defined volumes. A moderate correlation between FEV/FVC and functional volume was achieved by selecting an appropriate threshold for ventilation, perfusion, and matched function from both image sets. For ventilation imaging, the highest correlations were observed with FEV/FVC using 10–30% thresholds (*r* = 0.54–0.61). For perfusion imaging, similar correlation coefficients (*r* = 0.56–0.57) between FEV/FVC and functional volume were observed from 30 to 55% threshold values. For the matched V/Q volumes, the greatest correlations between FEV/FVC and functional volume observed was of the range 15–30% (*r* = 0.57–0.59). A weak correlation was observed with other lung function measures FEV, DLCO, and functional volume.

## Discussion

Radiopharmaceutical uptake globally throughout the lungs in ventilation/perfusion PET imaging is associated with overall lung function as measured by common PFT metrics. Whereas PFT can only define global lung function, assessment of regional lung function within anatomical subunits of the lung requires advanced imaging techniques. There is potential benefit in the ability to localise high- and low-functioning regions which may assist with surgical or radiotherapy planning. Previous work has investigated the correlation between FEV/FVC and an expert-defined manual contour of the functional lung as well as a semi-automated method [1, 2]. While the results of the automated method developed in this work are not as strongly correlated as the fully manual method (*r* = 0.78 and 0.81 for ventilation and perfusion, respectively), this study’s results were an improvement over the semi-automated method (*r* = 0.48 and 0.46) which involved defining a threshold at 15% of the maximum uptake inside the lung and manually removing regions of suspected clumping.

In principle, functioning lung tissue should exhibit both ventilation representing localised air flow and perfusion representing the ability to perform gas exchange to the blood. The matched functional volume, which includes the intersection of highly ventilated and perfused threshold regions, should be best correlated with pulmonary function tests. In this patient cohort, a range of threshold values applied to the reduced peak intensity shared similar correlations. Ventilation imaging alone had a limited range in which the correlation was relatively strong, this suggests that this ventilation technique may be more susceptible to variations in uptake distribution.

As a guide for potential threshold values in future applications, our observations suggest a range of 15–30% of adjusted peak uptake value for matched and ventilation imaging. For perfusion imaging, a broader range of thresholds (15–65%) offered a comparable correlation to lung function. Implementation of this methodology is proposed to be an addition to the typical workflow outlined: image acquisition, clinician assessment with raw imaging to determine the adequacy of the acquisition, and lastly automatically define regions of functional lung to provide volumes as supplementary information.

Ventilation imaging more frequently required a reduction in uptake maximum than perfusion imaging (48/52 and 21/66, respectively). A reduction in the uptake maximum is not a direct indication for the presence of tracer clumping—its intent is to provide a more representative peak value for the lungs that exhibit large heterogeneity of uptake. Additionally, it should be noted that when normalised volumes (0–3.34% ventilation, 0–4.88% perfusion) were excluded from the calculation of fractional lung function as opposed to being included, the resulting Pearson correlation remained relatively similar due to the relatively small magnitude of volume being normalised.

This methodology may be applied in future applications such as estimation of global lung function as a surrogate for conventional pulmonary function tests with added information on the regional distribution of function, lung avoidance in radiotherapy by providing contours as DICOM structures on the radiotherapy planning software, and planning of anatomical lung resections which may aid the surgeon in defining estimated loss of function after lobectomy, segmentectomy, and pneumonectomy in the context of resection of lung cancers. A core limitation of this study arises from its reliance on homogenous uptake in the global lung volume; perfusion imaging can exhibit an uptake gradient under influence of gravity [14], resulting in functional regions being excluded via this automated process. Although this gravitational effect may affect tracer pooling and distribution, the relationship between PFTs as a global measure and the automated estimation should be maintained. Additionally, the apparent pattern of tracer uptake is constrained by the resolving power of the PET imaging system which limits the ability to perceive potential regions of heterogeneous function due to the partial volume effect and respiratory motion. Future work may advance further by applying the suggested methodology to segmental lung regions rather than a global volume.

## Conclusion

The method outlined describes an automated functional lung volume delineation technique that advances on previous studies by automating an initialisation step to manage the potential presence of hotspots prior to thresholding. Implementation of this methodology has the potential to provide consistency for lung function assessment in serial imaging, more accurate inter and intra-patient comparisons, and an avoidance map for clinical interventions.

## Data Availability

Data is not appropriate to be shared.
